# Divergent macrophage responses to Influenza A virus and *Streptococcus pneumoniae*: co-infection drives bacterial dominance whereas superinfection favors viral priming

**DOI:** 10.3389/fimmu.2026.1729086

**Published:** 2026-03-04

**Authors:** Javier Arranz-Herrero, Jana Baranda, Sergio Rius-Rocabert, Mikel Moreno-Vadillo, Ivan Gonzalez-Ruiz, Alberto Miranda-Bedate, Elena Pinelli, Ines Inchausti-Moya, Sara Izpura-Luis, Vicent Tur-Planells, Paloma Reche, Paloma Fernandez, Yolanda Revilla, Gustavo Del Real, Adolfo García-Sastre, César B Gutiérrez-Martín, Jordi Ochando, Estanislao Nistal-Villan

**Affiliations:** 1 Transplant Immunology Unit, National Center of Microbiology, Instituto de Salud Carlos III, Madrid, Spain; 2 Microbiology Section, Dpto. Ciencias (CC), Farmacéuticas y de la Salud, Facultad de Farmacia, Universidad San Pablo-CEU, Madrid, Spain; 3 Instituto de Medicina Molecular Aplicada-Nemesio Díez (IMMA-ND), Facultad de Medicina, Universidad San Pablo-CEU, CEU Universities, Madrid, Spain; 4 Center for Infectious Disease Control, National Institute for Public Health and the Environment, Bilthoven, Netherlands; 5 Department of Microbiology, Icahn School of Medicine at Mount Sinai, New York, NY, United States; 6 Centro de Biología Molecular Severo Ochoa (CSIC-UAM), Universidad Autónoma de Madrid, Cantoblanco, Madrid, Spain; 7 Department of Biotechnology, National Institute of Agricultural and Food Research and Technology (INIA-CSIC), Madrid, Spain; 8 Global Health and Emerging Pathogens Institute, Icahn School of Medicine at Mount Sinai, New York, NY, United States; 9 Department of Medicine, Division of Infectious Diseases, Icahn School of Medicine at Mount Sinai, New York, NY, United States; 10 Department of Pathology, Molecular and Cell-Based Medicine, Icahn School of Medicine at Mount Sinai, New York, NY, United States; 11 The Tisch Cancer Institute, Icahn School of Medicine at Mount Sinai, New York, NY, United States; 12 The Icahn Genomics Institute, Icahn School of Medicine at Mount Sinai, New York, NY, United States; 13 Department of Animal Health, Faculty of Veterinary, Universidad de León, León, Spain; 14 Department of Oncological Sciences, Icahn School of Medicine at Mount Sinai, New York, NY, United States

**Keywords:** coinfection, *in vitro*, influenza, *Streptococcus*, superinfection, transcriptomics

## Abstract

**Introduction:**

Respiratory coinfections involving Influenza viruses, including Influenza A viruses (IAV) and bacteria, significantly worsen disease severity and remain a major public health concern, particularly during seasonal and pandemic flu outbreaks. Among bacterial pathogens, *Streptococcus pneumoniae* (Spn) and *Streptococcus suis* cause secondary infections in humans and swine respectively following influenza. The immunological mechanisms driving coinfection severity, especially the differences between simultaneous and sequential infections, are incompletely defined.

**Methods:**

We developed an *in vitro* differentiated bone marrow-derived macrophages (BMDMs) model to examine transcriptional and protein-level responses during IAV-Spn coinfection or sequential infection. BMDMs were infected with IAV and Spn either simultaneously or with a 48-hour delay.

**Results:**

RNA-Seq and OLINK proteomic analyses revealed that simultaneous coinfection elicits a synergistic inflammatory response similar to that caused by Spn alone, with strong activation of NF-κB-dependent genes. In sequential superinfection, responses were shaped by viral priming, with bacterial challenge further amplifying genes linked to inflammation and fibrin clot formation, potentially contributing to disease severity. These effects were consistent across different IAV subtypes when tested in combination with porcine *Streptococcus suis* serotypes that impose a comparable burden in pigs during influenza coinfection. Additionally, age is a determinant of BMDM responses. This model offers an advantageous tool for studying coinfection dynamics in human and veterinary medicine.

## Introduction

Human fatalities caused by Influenza viruses are a major concern in countries with advanced healthcare systems. It is estimated that influenza-related complications result in approximately 500,000 deaths worldwide, according to the World Health Organization (WHO). Around 20% of these influenza-associated deaths are linked to bacterial coinfections, with *Streptococcus pneumoniae* (Spn) being the most common (1). Similarly, Influenza A and *S. suis* are two of the main pathogens of the Porcine Respiratory Complex (PRC), which endemically affect pig herds worldwide and cause large economic losses and zoonotic risk (2).

Severe Influenza infections are strongly associated with exacerbated activation of the host’s inflammatory responses (3), characterized by high levels of inflammatory cytokines, chemokines, and acute-phase reactants (4). In uncontrolled situations, inflammatory responses may trigger deleterious mechanisms leading to cell death, contributing to immunopathologies and exacerbating disease severity (3).

Tissue resident alveolar macrophages (TR-AMs) form the frontline barrier in the lung that coordinates innate and adaptive responses against inhaled pathogens and pollutants. TR-AMs play a key role in initiating and resolving the immune response in the lungs (4). They self-renew but can also be replaced by recruited monocytes from the bone marrow that differentiate into inflammatory macrophages; such recruitment and replacement can reprogram the alveolar macrophage pool after injury or infection (5, 6). During acute infection, the bone marrow mobilizes neutrophils and monocytes, which enter the circulation and traffic to inflammation sites, and differentiate locally into macrophages shaped by tissue signals (7). Because bone marrow-derived precursors differentiated into these recruited macrophages, *in vitro* differentiation of BMDMs serves as a good model to study macrophage behavior in different infection settings (8). Macrophages display high phenotypic plasticity that is essential to their functions; distinct activation states emerge in response to cytokines, microbial ligands, and local signals, all of which determine effector programs (9, 10). Although macrophage activation *in vivo* represents a spectrum of functional phenotypes with intermediate or overlapping characteristics, M1 and M2 nomenclature is used to simplify macrophage function *in vitro* (8). Classically activated “M1−like” macrophages are pro−inflammatory and microbicidal, whereas alternatively activated “M2−like” macrophages favor resolution, tissue repair, and immunoregulation (11). Importantly, for inducing different functional macrophage programs, two cytokines are widely used: GM-CSF and M-CSF (10). GM-CSF (Granulocyte-Macrophage Colony-Stimulating Factor) is typically induced during inflammation and promotes pro-inflammatory responses and antigen-presentation (M1) while M-CSF (Macrophage Colony-Stimulating Factor) is constitutively expressed in circulation and supports homeostatic, survival, and repair phenotypes in tissue resident macrophages (M2) (12, 13). Similarly, alveolar macrophages in the lung polarize toward alternative activation states in response to changes, like the ones associated with acute infection inflammation within the local microenvironment (14).

The virulence factors of pathogens strongly shape disease severity and the macrophage responses (15). In coinfections with IAV and bacteria, timing (simultaneous *versus* sequential) and pathogen properties drive distinct clinical outcomes, yet the macrophage underlying mechanisms remain incompletely characterized. To address this gap, we developed an *in vitro* model to analyze BMDM responses, integrating the transcriptional and proteomic profiling to compare single IAV or Spn infections with simultaneous coinfection and a 48−hour sequential superinfection. Simultaneous coinfection drives a synergistic, bacterially dominated inflammatory transcriptional program, whereas sequential superinfection is primarily shaped by viral priming. These distinct macrophage programs vary with pathogen strain/serotype and with mouse age in our BMDM model, pointing to clinically relevant variables of heterogeneity that can modulate disease severity. This controlled macrophage infection system defines mechanistic readouts of host-pathogen interplay and highlights candidate pathways for targeted intervention in influenza-associated bacterial superinfections.

## Materials and methods

### Mice

C57BL/6J mice were purchased from the Jackson Laboratory, Bar Harbor, ME and maintained under specific pathogen-free conditions in accordance with institutional animal care ethics committee regulations at the Instituto de Salud Carlos III (ISCIII). All animal procedures were approved by the Ethics Committee for Animal Experimentation of the Instituto de Salud Carlos III and authorized by the competent authority (PROEX 021.6/22), in accordance with RD 53/2013 and Directive 2010/63/EU. Experimental groups contained equal numbers of male and female mice. PROEX covers animal colony maintenance and authorized sacrifice/euthanasia, with no prior experimental manipulation or intervention of the animals. Euthanasia was performed by gradual−fill CO_2_ inhalation (2.1–4.9 L/min; 30–70% chamber volume/min) in standard cages (~7−L volume) in accordance with AVMA guidelines and institutional approval, with death confirmed prior to tissue collection. Bone marrow was obtained post-mortem. No animals were subjected to experimental procedures prior to the sacrifice.

### Bone marrow-derived monocyte isolation and cell culture conditions

Murine bone marrow was harvested by flushing the humeri, femur, and tibia with sterile PBS (Gibco, Billings, MT, USA), and the cell suspension was filtered through a 70 μm cell strainer (Corning Falcon, USA). Bone marrow cell suspension was centrifugated at 1500rpm (24 x 1.5/2mL rotor - MA-2024) 4°C for 5 min and resuspended in RPMI 1640 (1X) + GlutaMAX™ (Gibco, Walthman, MA, USA), supplemented with 10% Fetal Bovine Serum (FBS) (Gibco), 1% of Penicillin-Streptomycin (Gibco, Walthman, MA, USA), 1% HEPES (Gibco, Walthman, MA, USA), 1% Sodium Pyruvate (Gibco, Walthman, MA, USA) and β-mercaptoethanol 100 µM. For macrophage differentiation, bone marrow cells were plated at 4x10^6^ cells/ml in 12-well plates and cultured in medium containing either recombinant murine GM-CSF (25ng/mL; Gibco, Waltham, MA, USA) or M-CSF cytokines (50ng/mL; PeproTech, Cranbury, NJ, USA). BMDM were cultured for 7 days with a medium change on day 3 with fresh supplemented GM-CSF/M-CSF medium to complete macrophage differentiation. Differentiation was confirmed by morphology (Leica DFC300 FX camera), and BMDM were recovered using Accutase (*Gibco*) under manufacturer instructions, and purity was confirmed by flow cytometry using LSRFortessa flow cytometer (BD Biosciences, USA).

### Porcine alveolar macrophages

Porcine alveolar macrophages (PAMs) were obtained by bronchoalveolar lavage as previously described by Carrascosa and colleagues (1982) (16). After collecting PAMs were cryopreserved in porcine serum containing 10% DMSO. Thawing was carried out rapidly at 37 °C, followed immediately by gentle washing in pre−warmed medium before cell plating. PAMs were cultured in Dulbecco’s Modified Eagle Medium (DMEM Gibco) supplemented with 2 mM L−glutamine, 0.4 mM non−essential amino acids, 100 U/mL gentamicin, and 10% porcine serum obtained from the same donor animals from which the cells were isolated.

Cells were allowed to adhere and recover for 24 h before the initial inoculation with infectious agents. All infections were performed under the same atmospheric and temperature conditions used for maintenance (37 °C, 5% CO_2_). Inoculation procedures, incubation times, and sample harvesting were conducted in full parallel with the protocols described for the murine BMDM experiments, ensuring methodological consistency.

### Virus culture and titration

Influenza A/PR/8/34 (H1N1) virus was grown in the allantoic fluid of 8-day-old embryonated chicken eggs. After two days of incubation at 37°C, the allantoid fluid was extracted, centrifuged (1,000 x g for 10 min at 4 °C), aliquoted, and stored at -80°C. Viral stocks were titrated by plaque assay on Madin-Darby canine kidney (MDCK) cells (ATCC^®^ CRL-3216) on p12 plates. MDCK cells were maintained in DMEM (Gibco) supplemented with 10% FBS (Gibco) and 1% penicillin/streptomycin at 37°C, 5% CO_2_. For coinfection and superinfection experiments, we used a multiplicity of infection (MOI) of 1 for IAV. Additional Influenza A subtypes (H1N1, H1N2, H3N1, and H3N2) used in comparative experiments were obtained from pigs (isolated by Gustavo del Real group) and were grown and tittered as described above. In all experiments, a single IAV subtype was used per plate/assay. No pooling of Influenza subtypes occurred within any well or experiment. When swine IAVs (H1N1, H1N2, H3N1, H3N2) were compared, they were run in independent, parallel experiments and analyzed against their own single−virus controls. Virus−free medium used in mock conditions was identical to that used for viral dilutions to ensure handling−matched controls.

### *Bacterial* maintenance and titration

*Streptococcus pneumoniae* serotype 1 (ATCC^®^ 6301) was grown on blood agar plates at 37 °C with 5% CO_2_. Fresh cultures were started by streaking a single colony onto a new blood agar plate the day before infection and incubating overnight. Bacterial colonies were suspended in 5mL of sterile saline solution and adjusted to 0.5 McFarland units (McF) using a nephelometer (Densimat). Serial dilutions plating in Tryptone Soy Agar with 5% Sheep Blood (Thermo Scientific) was used to confirm viable counts, and 0.5 McF of *S. pneumoniae* (Spn, ATCC^®^ 6301) corresponded to ~3.36x10^7^ CFU/mL. For all coinfection and superinfection protocols, 0.5 McF of the freshly grown bacterial inoculum was used as an MOI of 1. The different *Streptococcus suis* utilized in the study are indicated in **Supplementary Table 1**. Bacterial infections were performed individually. *S. pneumoniae* and *S. suis* were never co−inoculated. When different *S. suis* serotypes were evaluated, each serotype was assayed in a separate arm, with volume−matched virus−free mock controls. Bacteria-free medium used for mock conditions matched the medium used to suspend bacterial inocula.

### Coinfection assay

On day 7 post-differentiation, BMDMs were washed three times with sterile PBS to remove any residual serum and cytokines, and infection was performed in RPMI culture media without antibiotics. Cells were inoculated either with Influenza A virus (IAV) alone, or with the bacteria at MOI = 1 alone (or mock), or with both pathogens simultaneously (coinfection). Coinfection experiments therefore included four parallel conditions: mock infection; single IAV infection with the corresponding volume of bacterial medium without bacteria; single Spn infection with the corresponding volume of viral medium without virus; and simultaneous IAV + Spn infection. For simultaneous coinfection, the viral and bacterial inocula were added at the same time and incubated for 8h at 37 °C with 5% CO_2_. After incubation, culture supernatants were collected for proteomic analysis and cells were lysed in TRIzol ^®^ (Thermo Scientific, Waltham, MA, USA) for RNA extraction, all stored at -80°C. No mixtures of Influenza subtypes or bacterial species were used within any well. Supernatants and RNA were collected after 8h in all arms. “Mock” refers to cells exposed to the same volume of virus-free or bacteria-free medium used to prepare inocula, following identical adsorption, washing, and incubation steps as infected cultures, but without the pathogen.

### Superinfection assay

Similar to the coinfection protocol, on day 7 BMDMs were washed and exposed for 1 h either to Influenza A virus (one subtype at the time of IAV; MOI = 1) or to a mock viral inoculum consisting of the same volume of virus-free medium. Mock viral inoculum consisted of the same volume of virus−free medium, ensuring identical handling without the pathogen. After this 1h adsorption, fresh antibiotic-free RPMI was added and cultures were incubated for 48h. At 48h, cells received either a mock bacterial inoculum (bacteria-free medium, volume-matched) or freshly prepared bacteria (one species at the time) from overnight blood-agar cultures, added directly to the wells at MOI = 1 (adjusted to macrophage counts), and were incubated for an additional 8h. Mock bacterial inoculum consisted of bacteria-free medium at the same volume as bacterial suspensions. Thus, the superinfection experiments consisted of two sequential phases, a 48h priming period with either mock or IAV, followed in each case by an 8h secondary challenge with either mock or bacteria. Supernatants were collected and cells lysed in TRIzol ^®^ at the end of these 8h, except for the 48h mock control, stored at -80°C for downstream applications.

### RNA extraction

Total RNA was extracted from cell lysates using TRIzol ^®^ followed by chloroform phase separation (PanReac AppliChem ITW reagents, Castellar del Vallès, Catalonia, Spain) according to manufacturer’s protocol. Extracted RNA was treated with RNase-free DNAse I (Qiagen, Hilden, NRW, Germany) to remove genomic DNA following the manufacturer’s instructions. RNA concentration and purity were measured by NanoDrop spectrophotometry (Thermo Scientific, Waltham, MA, USA), and samples were stored at -80°C until downstream use.

### RNA sequencing, analysis, and data processing

RNA quantity and purity were measured by Lab-on-Chip analysis using an Agilent 2100 Bioanalyzer (Agilent Technologies, USA). Only samples with RNA Integrity Number (RIN) ≥8 were used for library preparation. TruSeq Stranded mRNA Library Prep Kit (Illumina, San Diego, California, USA) was used, with 500 ng of total RNA as input. Half reaction volumes were used through the Ilumina protocol. Sequencing was conducted with the Illumina NextSeq 500/550 High Output Kit v2.5 (single-end, 75 cycles). Samples were randomized and processed across four sequencing runs. Basecalling and demultiplexing were carried out using bcl2fastq2 Conversion Software v2.20, generating demultiplexed FASTQ files based on sample-specific barcodes (>15 million reads per sample).

Quality of the reads was assessed using FASTQC software (https://www.bioinformatics.babraham.ac.uk/projects/fastqc/), processed with Fastp and aligned to the *Mus musculus* (GRCm38) and most recent transcript annotations using kallisto (v0.46.1) (17). Raw counts were filtered for low-count genes, excluding targets with less than 1 count in at least 4 samples. Filtered count distribution was normalized with the DESeq R package using *DESeqDataSetFromMatrix()* and *DESeq()* functions.

The differential analysis was performed with DeSeq2 R package (18) under standard parameters. Genes were considered significantly expressed if they showed an adjusted p-value (Bonferroni-Hochberg multiple testing, *padj*) lower than 0.05 and a fold change > 2.

Heatmaps were plotted using *pheatmap()* function from *pheatmap* and *ComplexHeatmap* R libraries (19). Dendrogram trees were built by the average clustering method, obtaining gene distances by *Spearman* correlation.

Gene Set Enrichment analysis (GSEA) and Gene Ontology (GO) enrichment were performed using *fgsea()* and the *enrichGO()* functions of the *fgsea* and *clusterProfiler* R packages, respectively (20). Finally, regulon analysis of transcription factors was performed using *DoRothEA* R package (21–23) under standard parameters.

### Relative quantification by RT-qPCR

cDNA was synthesized from 1µg total RNA by using a high-capacity cDNA Reverse Transcription Kit (Applied Biosystems, Carlsbad, CA, USA) following the manufacturer’s instructions. Quantitative PCR (qPCR) was performed with SYBR Premix Ex Taq (Takara Kusatsu, Shiga, Japan) following the manufacturer’s instructions on a 7900HT fast real-time PCR system (Thermo Scientific, Waltham, MA, USA) using the primers needed to quantify the expression of the genes under analysis. Relative expression was calculated by the ΔΔCt method and normalized to appropriate housekeeping genes. Primer list has been included as a **Supplementary Table 2** (24–27).

### Olink proteomic analysis

Supernatants from coinfection and superinfection assays were analyzed using the Mouse Olink^®^ Target 96 Inflammation Cytokine panel (Olink Proteomics AB, Uppsala, Sweden) following the manufacturer’s instructions and performed by the Olink group at the Institute of Applied Molecular Medicine (IMMA), Universidad San Pablo CEU. Results from the analyzed protein biomarkers were then provided in Normalized Protein eXpression (NPX) units. Further statistical analyses were carried out in-house using R software to evaluate the protein expression profiles across conditions and to perform comparisons between samples.

### Statistical analysis

Statistical details for each specific experiment are described in each figure legend. Data for all experiments represent the geometric mean and the standard deviation (SD). Statistical significance was determined by *p ≤ 0.05, **p ≤ 0.01, ***p ≤0.005, **** p ≤ 0.001. Statistical analyses were conducted using GraphPad Prism v9.1 software.

## Results

### Influenza coinfection with *Streptococcus pneumoniae* increases gene expression of NF-κB-pathway dependent genes, as well as DNA repair and cell survival pathways

**Figure 1:**
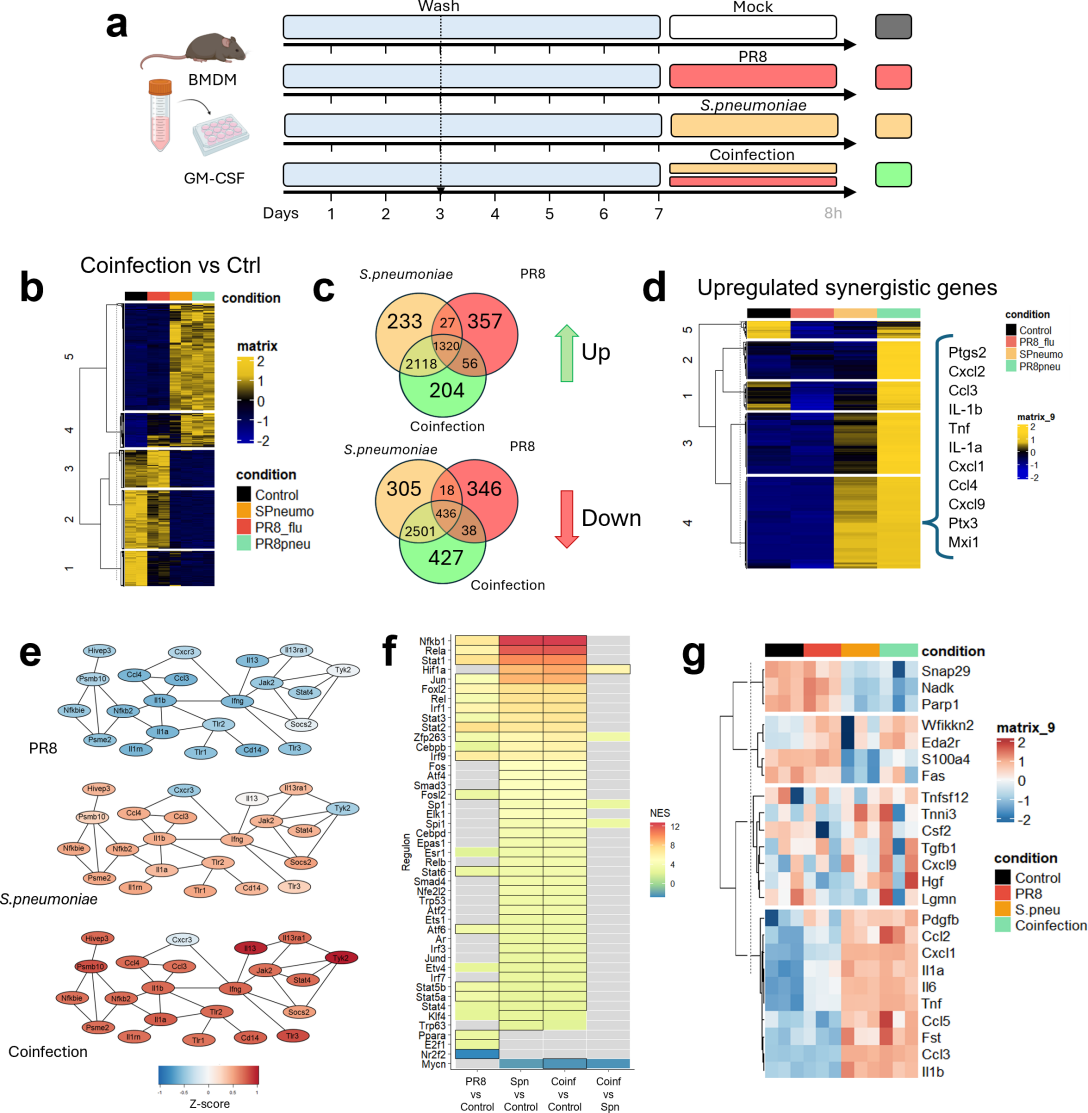
*In vitro* coinfection of PR8 and Spn follows bacterial transcriptional pattern and increased inflammation via NF-κB pathway, cytokine production, and chemokine release. **(a)** Workflow scheme for the *in vitro* coinfection model. Murine BMDM were differentiated with GM-CSF for 7 days. Cells were then infected under four parallel conditions: mock, single PR8 infection (with volume-matched bacteria-free medium), single Spn infection (with volume-matched virus-free medium), or simultaneous PR8 + Spn coinfection. Supernatants were collected for Olink; RNA was extracted and used for RNAseq. Panel created with Biorender.com. **(b)** Heatmap of all significant regulated genes obtained by RNASeq sequencing after comparing the coinfection condition vs the control using the DESeq2 pipeline. **(c)** Venn diagrams of all significant genes in each pairwise comparison vs the control. The top of the panel shows upregulated genes, and the bottom of the panel shows downregulated genes. **(d)** Clustered Heatmap of synergistic genes: genes whose average expression in the coinfection condition exceeded the sum of the average counts in the Spn condition plus 1.5 times the average counts in the PR8 condition. Interesting genes from cluster 4 are shown in the panel. **(e)** Functional protein association network of the main upregulated pathway with synergistically upregulated genes was performed using the STRING database. Colors of the nodes represent the z-score value. **(f)** Potential transcription factor usage by Normalized Enrichment Score (NES) of regulon activation obtained with *DoRothEA*. Colored tiles indicate genes with FDR < 0.15, while black-bordered tiles highlight those with FDR < 0.05. **(g)** Heatmap displaying all proteins from the Olink mouse panel with values over the lower the detection limit, clustered into 4 main groups. Group 4 shows upregulated proteins in coinfection. One IAV subtype per experiment; no pooling of Influenza subtypes, and no bacterial species mixtures.

To model and investigate host inflammatory responses during coinfection, GM-CSF-differentiated BMDMs were exposed *in vitro* to individual or simultaneous infection with both Influenza A and Spn for 8h as described in materials and methods. An overview of the experiment can be shown in **Figure 1a**.

Extracted RNA was used for Illumina sequencing as described in the methods section. The DESeq2 pipeline was used for pairwise comparisons, and significant genes for coinfection *versus* control (**Figure 1b**) showed that modulation of transcription in coinfection follows a pattern similar to bacterial infection. A Venn Diagram comparing all significant genes for all pairwise comparisons vs control showed a total of 1320 genes significantly increased in all conditions vs control (**Figure 1c**). Also, 2118 genes were shared between coinfection and Spn conditions. Influenza-infected BMDMs showed 1760 significantly upregulated genes, but only 56 were shared with coinfection and 27 with Spn conditions individually, and 357 specific genes for Influenza virus infection. Regarding downregulated genes, the same pattern is observed. Most of the genes are shared between coinfection and Spn conditions: 2501 are exclusively shared by both conditions, and 436 are shared by all conditions. Among downregulated genes, 427 were exclusive to coinfection, 305 to Spn, and 346 to PR8 Influenza virus.

Further analysis was performed to look for synergistically expressed genes in coinfection, as these genes might serve as indicators associated with exacerbated inflammation. For this, we applied a stepwise filtering strategy. First, we selected genes showing significant differential expression compared to the control in at least one experimental condition. Genes with zero counts across all conditions were subsequently removed. To define synergistic expression, we considered genes whose average expression in the coinfection condition exceeded the sum of the average counts in the Spn condition plus 1.5 times the average counts in the PR8 condition, a threshold chosen to capture responses beyond additive effects. The resulting genes were plotted in a clustered heatmap (**Figure 1d**), showing 5 major clusters. Of these, clusters 2–4 displayed genes markedly enriched in the coinfection setting (**Supplementary Figure 1a**). Specifically, cluster 2 comprises 131 genes uniquely elevated in coinfection, such as *Fcgr4* or *Il3ra*. Cluster 3 included genes strongly induced by Spn alone, but even more pronounced during coinfection, such as *Saa3* or *Batf2*. Cluster 4 contained genes already elevated under Spn infection yet further upregulated under coinfection. Full list of synergistic genes in coinfection can be found in **Supplementary Data 1**, and downregulated genes in **Supplementary Data 2**.

Gene Ontology (GO) and KEGG pathways (**Supplementary Figure 1b**) for the gene lists in the three clusters (2, 3 and 4) show a relationship between these genes and a potential high cytokine and chemokine activity, related to TNF signaling via the NF-κB pathway. Also, transcriptional inflammatory responses, including IL-6/JAK/STAT3 signaling pathway, are significantly activated. Among the genes associated with cytokine activity and inflammatory processes, *ptgs2*, *cxcl2*, *ccl3*, *il-1β, tnfα, il-1α, cxcl1, ccl4, cxcl9, ptx3*, or *mx1* were found in cluster 4. These genes, predominantly induced by bacterial infection and further amplified during coinfection, exhibited some of the highest and most significant Log_2_FC (**Supplementary Figure 1c**). The Gene Set Enrichment Analysis (GSEA) for coinfection vs Spn condition (**Supplementary Figure 1d**) also showed a significant increase in TNF signaling via the NF-κB pathway, besides other pathways related to DNA repair and cell survival.

Protein–protein interaction analysis with StringR, using a high-confidence threshold (interaction score = 0.900), revealed an upregulation of the NF-κB pathway. StringR integrates data from multiple sources to construct interaction networks, thereby providing insights into how coinfection may potentiate coordinated immune signaling within this pathway (**Figure 1e**). In parallel, transcription factor activity was inferred using the *DoRothEA* R package, calculating Normalized Enrichment Scores (NES) for regulon activity based on gene expression data. This analysis further confirmed NF-κB1 activation in both Spn and coinfection conditions compared to control, while hypoxia-inducible factor 1-alpha (*HIF-1α*) dependent signaling exhibited significant activation specifically in coinfection vs Spn, indicating a distinct regulatory signature characteristic low oxygen consumption and switching on metabolism, and survival genes (**Figure 1f**).

A second approach to identify synergistically expressed genes was performed by analyzing variations in Log_2_FC and Pearson correlation between coinfection and Spn conditions. The difference (dr) between the mean Log_2_FC_Coinfection_ and Log_2_FC_Spn_ was calculated here. Genes significant in both conditions with a difference > 0.2 Log_2_FC_dr_, were considered upregulated, while those with a difference < -0.2 Log_2_FC_dr_ were considered downregulated. A linear correlation plot comparing both conditions is shown in **Supplementary Figure 1e**, where upregulated genes are highlighted in red, and downregulated genes, in blue. Although the gene lists obtained for this method varied slightly from the first method, the GO and KEGG enrichment analyses of synergistic genes from this second method showed similar results (Data not shown).

Protein expression at 8h was also measured by Olink^®^ relative quantification proteomic panel. Principal components analysis (PCA) showed comparable normalized protein expression (NPX) profiles in Spn and coinfection conditions for this panel of proteins (**Supplementary Figure 1f**). Subsequent analysis demonstrated a pronounced enrichment of chemokines and interleukins in both conditions (**Figure 1g**). Notably, Follistatin (fst) or chemokines such as CCL2, CCL5 were particularly elevated in Spn and coinfection. Full list of NPX of proteins in coinfection can be found in **Supplementary Data 3**.

### Influenza strongly shapes host gene expression in superinfections

**Figure 2:**
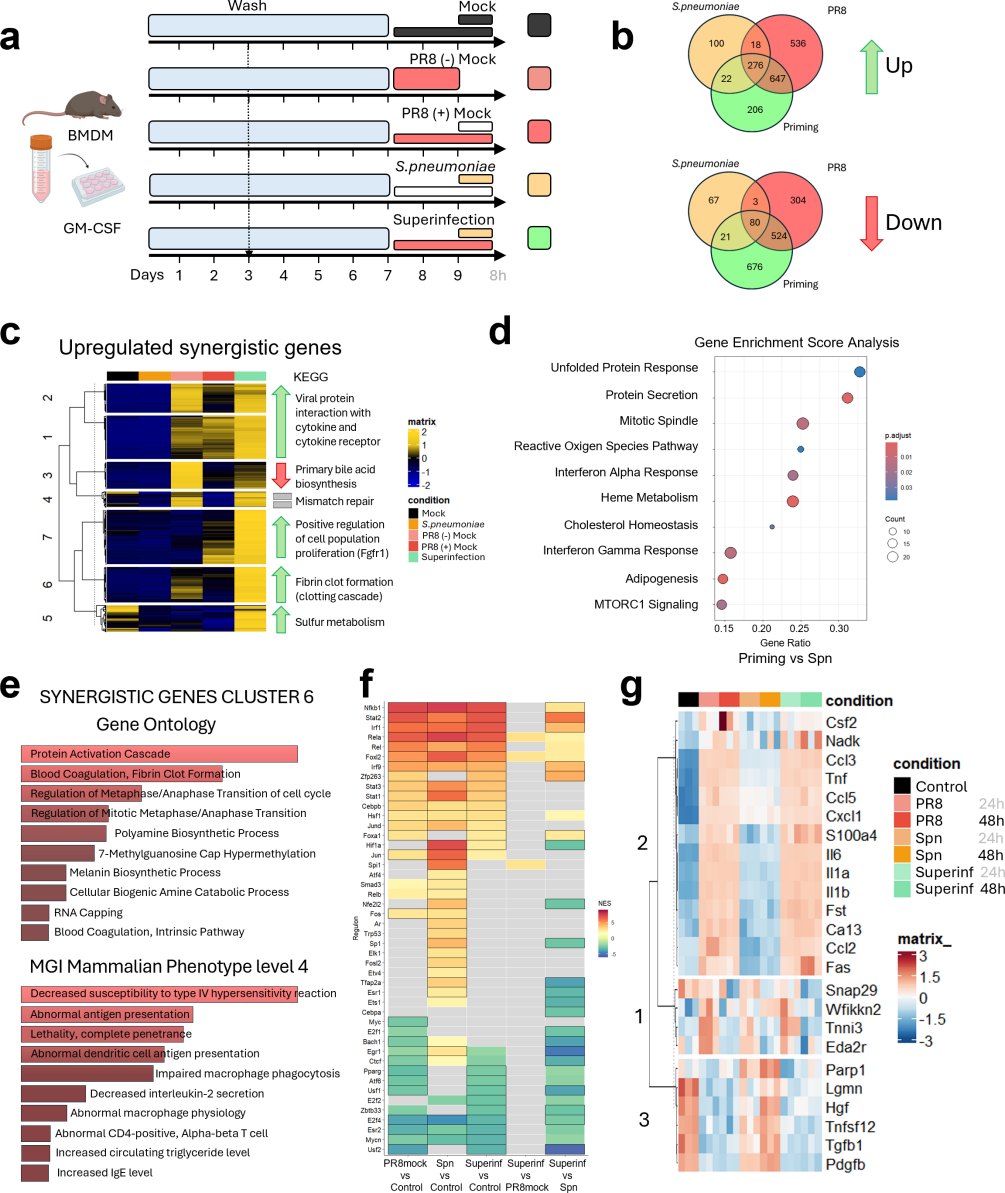
Transcription regulation of *in vitro* superinfection with Influenza virus and *Streptococcus pneumoniae* is determined by prior Influenza virus infection. **(a)** Workflow scheme for the *in vitro* superinfection model. All assays begin with bone marrow extraction from adult mice, and cell differentiation with GM-CSF cytokine for 7 days. BMDMs were first exposed for 1 h to either PR8 (MOI = 1) or a mock viral inoculum, then incubated for 48 h in antibiotic-free medium. After this priming phase, cells received either a mock bacterial inoculum or freshly prepared Spn at MOI = 1 for 8 h, after which supernatants and cell lysates were collected for Olink proteomics and RNA analysis. Panel created with Biorender.com. **(b)** Venn Diagrams of all significant genes in each pairwise comparison versus the control. The top of the panel shows upregulated genes, and the bottom of the panel shows downregulated genes. **(c)** Clustered heatmap of synergistic genes. KEGG pathways for each cluster are shown in the panel. **(d)** Barplots of top gene ontology (GO) and MGI mammalian phenotype level 4 molecular signatures of genes from primed genes in cluster 6. **(e)** GSEA analysis plot displaying top 10 significantly upregulated pathways in superinfection condition compared to the Spn-infected conditions. **(f)** Normalized enrichment score (NES) of regulon activation obtained with *DoRothEA*. Colored tiles indicate genes with FDR < 0.15, while black-bordered tiles highlight those with FDR < 0.05. **(g)** Heatmap displaying all proteins from the Olink mouse panel with values over the lower detection limit, clustered into 3 main groups. Group 2 shows upregulated proteins in Influenza-infected conditions. Superinfection was strictly sequential (IAV for 48 h followed by bacteria); one virus subtype and one bacterial species per experiment; sampling occurred only after the 8 h secondary step. Mock = pathogen−free medium control; single−infection controls = IAV−only or Spn−only.

To study the macrophage response to a sequential infection, we developed a second *in vitro* model, where bacterial exposure occurs following an initial Influenza infection, simulating a staggered infection sequence. This will be named from now on as superinfection. For these superinfection experiments, differentiated BMDMs during 7 days with GM-CSF were infected with Influenza PR8 (MOI = 1) or mock-infected for 24 or 48 hours. After that time, Spn was added at MOI = 1 for 8h, followed by RNA extraction and the transcriptomic analysis procedures described in materials and methods. A detailed schematic representation of all conditions is shown in **Figure 2a**. Throughout all superinfection experiments, “mock” denotes handling-matched inoculations with pathogen-free medium, whereas “single-infection controls” designate cultures infected with only IAV or only bacteria.

DESeq2 pipeline was used, as mentioned above, for differential transcriptomic expression analysis. In this experiment, the three conditions, superinfection, Spn, and PR8, present 276 upregulated and 80 downregulated differentially expressed genes (DEGs) (**Figure 2b**) compared to the mock control. Interestingly, when comparing between groups, most superinfection DEGs are only shared with the PR8 condition (647 upregulated and 524 downregulated DEGs) compared to those shared with Spn (22 upregulated and 21 downregulated). Of the 1151 upregulated genes for superinfection, 206 were exclusive to that condition when compared to the control. 536 genes were unique for PR8 infection, and 100 for Spn infection. On the contrary, 676 out of 1301 downregulated genes were only found in the superinfection condition, 304 out of 911 for PR8, and 67 out of 171 for Spn.

Differential expression analysis revealed that, although bacterial infection triggered inflammatory responses like those observed in coinfection, prior viral exposure exerted a strong priming effect on gene regulation after 48h. Consequently, induction of the gene expression during superinfection was largely shaped by the preceding viral infection. Both PCA and Pearson correlation analyses (**Supplementary Figure 2a**) supported this effect, with component 1 explaining 68.1% of the variance and clearly separating the experimental groups.

To define synergistically upregulated genes under superinfection, we applied the same strategy as for coinfection, but with the order of conditions reversed: genes were considered synergistic when their average expression in the superinfection condition exceeded the sum of the average counts in the PR8 condition plus 1.5 times the average counts in the Spn condition. This threshold was again used to capture responses beyond additive effects. The resulting gene set (**Figure 2c**) clustered into seven major groups according to KEGG annotation, including gene expression patterns related to viral protein interaction with cytokines and cytokine receptors, positive regulation of cell proliferation, and fibrin clot formation, all of which were strongly primed in the superinfection condition. Full list of synergistic genes in Superinfection can be found in **Supplementary Data 4**, and downregulated genes in **Supplementary Data 5**.

GO enrichment of synergistically expressed genes highlighted inflammatory cell recruitment and regulation (**Supplementary Figure 2b**), even though the Influenza (48h) mock condition already showed an enrichment in cytokine-related pathways (**Supplementary Figure 2c**). Specifically, chemotaxis and migration of natural killer cells, eosinophils, lymphocytes, macrophages, monocytes, neutrophils, and granulocytes appeared to be regulated by inducted gene expression of chemokines such as *Ccl3*, *Ccl4*, *Ccl5*, *Ccl17*, *Ccl22*, and *Cxcl10*. Synergistic gene induction also regulated pathways associated with Type I IFN responses, negative regulation of viral transcription, and fibrinogenesis-related processes.

Analysis of the transcripts with additional molecular signature databases such as mammalian phenotype Level 4 from MGI (Mouse Genome Informatic), showed that top-upregulated genes under the superinfection condition were associated to other gene ontologies: abnormal macrophage physiology, hemolytic anemia, amyloidosis, decreased susceptibility to type IV hypersensitivity reaction, abnormal antigen presentation and increased susceptibility to *Orthomyxoviridae* infection inducing morbidity/mortality (data not shown).

Focusing on cluster 6, which includes strictly primed genes, GO showed enrichment in signaling pathways related to blood coagulation, regulation of mitosis, and RNA capping (**Figure 2e**). The phenotype signatures described for those sets of genes correlated with abnormal antigen presentation, lethality, impaired macrophage phagocytosis, and decreased susceptibility to type IV hypersensitivity.

GSEA of both DEGs and non-DEGs across individual pairwise comparisons revealed that prior Influenza infection did not result in significantly upregulated or downregulated pathways when compared with other Influenza-infected conditions. In contrast, while Spn alone exhibited a strong proinflammatory profile, consistent with the coinfection profile in **Figure 1**, prior influenza priming was associated with a marked Type I IFN signature, enhanced IFN-γ response, increased protein secretion, and unfolded protein response (UPR) activation (**Figure 2d**).

To validate the synergistic effect using an alternative approach, we applied the same Log_2_FC–Pearson correlation strategy used for the coinfection analysis, this time comparing the superinfection and the PR8mock infected (PR8 48h+8h) conditions. Genes exhibiting a Log_2_FC difference > 0.2 were classified as upregulated, while those with a difference < –0.2 were classified as downregulated (**Supplementary Figure 2d**). This analysis identified 358 upregulated genes, mainly associated with cytoplasmic translation, peptide and macromolecular biosynthesis, and chemokine receptor activity. Since these pathways closely overlapped with those identified by the first method, detailed results are not shown.

Transcription factor activity was also assessed using the *DoRothEA* framework (**Figure 2f**). In both viral and bacterial infections compared to control, the main regulons identified were *Nfkb1, Stat2, Irf1*, and *Rela*, consistent with the transcriptional profiles observed in the coinfection experiment. These findings indicate that activation of the NF-κB axis represents a common regulatory feature across coinfection and superinfection conditions. Notably, the comparison between superinfection and viral infection alone highlighted differences in *Rela, Foxl2*, and *Spi1*, suggesting additional layers of transcriptional regulation specific to the superinfection setting.

RNASeq results were validated by Olink^®^ proteomic profiling of the matched sample supernatants. PCA analysis (**Supplementary Figure 2e**) clustered bacterial 24h and 48h infection conditions together, whereas the superinfection condition aligned more closely with the corresponding Influenza-infected samples (24h or 48h). Component 1 accounted for 65.91% of the variance and clearly separated these groups from Spn and control samples. Normalized protein expression (NPX) analysis showed the elevated concentrations of Il6, Il-1α, Il-1β, Ccl2, Ccl3, Ccl5, Cxcl1, or TNF both in superinfection and Influenza-infected conditions (**Figure 2g**). Although bacterial infections alone also induced the expression of these proteins compared with control, their concentrations remained significantly lower than those observed in Influenza or primed (superinfection) conditions. Full list of NPX of proteins in superinfection can be found in **Supplementary Data 6**.

### *Streptococcus* species and serotypes cause heterogeneous immune responses in BMDMs

To validate the transcriptomic findings and further investigate pathogen-specific outcomes under distinct experimental conditions, the expression of key cytokine genes was analyzed by RT-qPCR in independent experiments.

**Figure 3:**
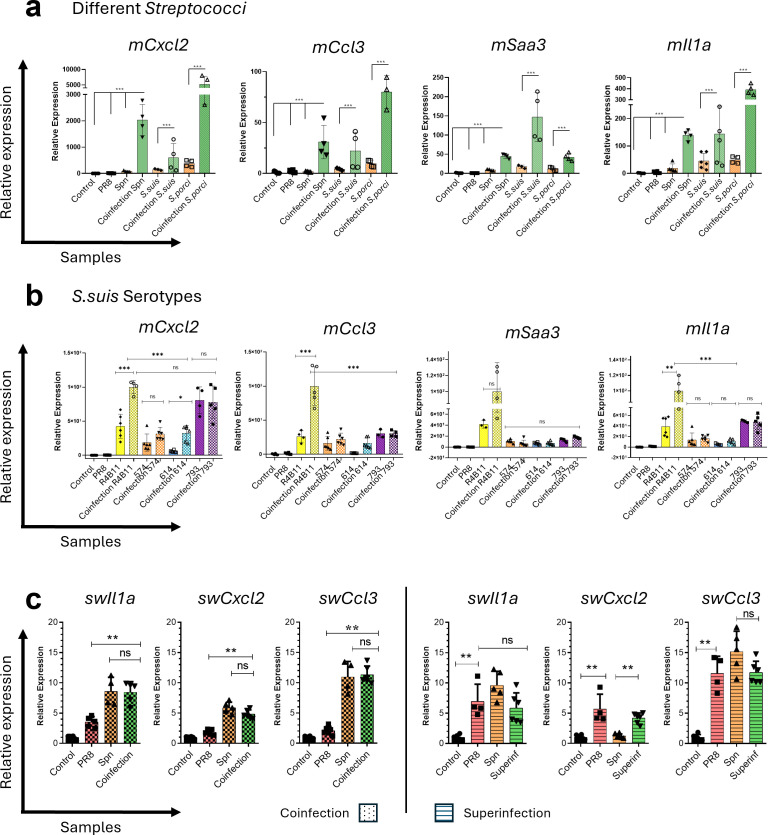
Gene expression in BMDMs during IAV–bacterial coinfection can be shaped by Streptococcus species and bacterial serotypes. **(a)** BMDMs were infected under four parallel conditions: mock, single IAV infection (with volume−matched bacteria−free medium), single bacterial infection (*S. suis*, *S. porci*, or Spn ATCC^®^ 6301 as reference, each tested separately with volume−matched virus−free medium), or simultaneous IAV + bacteria coinfection. *Cxcl2*, *Ccl3*, *Saa3*, and *Il-1α* relative gene expression were analyzed by RT-qPCR. **(b)** BMDMs infected or coinfected with *Streptococcus suis* clinical isolates from the lungs or brain of pigs. *Cxcl2*, *Ccl3*, *Saa3*, and *IL-1α* relative gene expressions were analyzed by RT-qPCR. **(c)** Primary porcine alveolar macrophages (PAMs) subjected to the same infection designs used for murine BMDMs: mock, single IAV, single *S. pneumoniae* (Spn), simultaneous IAV + Spn coinfection, and sequential IAV followed by Spn superinfection. Swine-specific *Il1a*, *Cxcl2*, *Ccl3*, as well as housekeeping genes expression were analyzed by RT-qPCR. Statistical significance was assessed by one-way ANOVA. *p ≤ 0.05, **p ≤ 0.01, ***p ≤ 0.001. Mock conditions consisted of pathogen−free medium applied with the same timing and handling as infected wells.

Given that swine, like humans, are prone to secondary infections by different *Streptococcus* species during Influenza episodes (28), we further extended our model to include additional clinically relevant species. In this experiment, coinfections involving PR8 and *S. pneumoniae* ATCC 6301 were compared with coinfections using PR8 and veterinary isolates of *Streptococcus suis* and *S.porci* (**Figure 3a**). Under identical infection protocols, all three *Streptococcus* species induced synergistic gene expression of *Il1a*, *Cxcl2*, *Ccl3*, and *Saa3*, standing out as the most consistently upregulated genes. While the magnitude of induction varied across species, *S. porci* elicited higher expression of *Il1a, Cxcl2*, and *Ccl3*, whereas *S. suis* showed a more pronounced upregulation of the Serum Amyloid A3 gene (*Saa3*), part of the serum amyloid A family of acute−phase proteins.

To further explore the variability of the synergistic response across different bacteria of the same species, we examined the response to coinfection across different *Streptococcus suis* serotypes obtained from swine lungs (serotypes 12 and 18) or brain (serotypes 1, 2, and 9).

Based on these observations, a subset of representative isolates was selected for more detailed analysis, including strain 574 (serotype 12, lung), strain 614 (serotype 2, brain), and strain 793 (serotype 9, brain) (**Figure 3b**). RT-qPCR analysis of Cxcl2, Ccl3, Saa3, and Il1a gene expression revealed that strain 793 elicited the strongest inflammatory response, with high induction of Cxcl2, Ccl3, and Il1a both when it was used to infect only with bacteria or during the coinfection with PR8. In contrast, strains 574 and 614 displayed more modest responses, with strain 614 showing a specific increase in Cxcl2.

Although we previously found that the immune response in coinfection is mainly driven by the bacterial component, we also tested whether different swine Influenza subtypes altered this outcome (**Supplementary Figure 3b**). Swine IAVs H1N1, H1N2, H3N1, and H3N2 were evaluated alone or in coinfection with *S. suis* R4B11 in BMDMs. Overall, all swine IAV subtypes induced lower inflammatory gene expression compared to the mouse-adapted PR8 strain. In coinfection, these viral strains did not markedly enhance cytokine induction beyond that observed with bacteria alone.

To address species specificity, we evaluated the same infection designs in primary porcine alveolar macrophages (PAMs). In coinfection, PAM transcriptional responses overlapped with those of Spn alone, consistent with bacterial dominance of early inflammatory signaling. In superinfection, we observed a mixed, primed profile: *Il1a* and *Cxcl2* reached peak levels comparable to IAV only or Spn only conditions, whereas *Ccl3* levels aligned with IAV only, indicating that prior viral exposure shapes chemokine output upon subsequent bacterial challenge (**Figure 3c**).

### Age, but not the initial activation state, is a differential factor determining BMDM response during coinfections and superinfections

From a biological standpoint, aging is characterized by a gradual decline in homeostatic balance, resulting in functional decline and an increased vulnerability to mortality (29, 30). Recent studies have shown that aging is associated with a progressive deterioration of immune competence, also known as immunosenescence, compromising both innate and adaptive immunity responses (31). This dysregulated response contributes to increased susceptibility and altered outcomes during viral infections and is partially driven by the dysfunction within the myeloid lineage, particularly affecting macrophage activity (32).

Macrophages can modify their gene expression and activation states in response to various signals, including growth factors and microbial stimuli. In the lung, proinflammatory M1 macrophages and tissue-healing M2 macrophages coexist during infection, with M1 cells usually contributing to inflammation and tissue damage, and M2 resident macrophages supporting tissue repair (14, 33). To determine whether these activation states affect the response to coinfection and superinfection, we first compared GM-CSF-derived M1-like and M-CSF-derived M2-like BMDMs’ transcriptional responses in the coinfection model. Despite their different differentiation pathways, macrophages generated with M-CSF were coinfected or superinfected (primed) with PR8 and Spn following the protocol used for **Figures 1** and **2**. The RT-qPCR analysis of selected genes exhibited similar induction patterns (**Supplementary Figure 4**) in M-CSF-differentiated BMDM compared to GM-CSF-differentiated BMDM (**Figure 3**).

**Figure 4:**
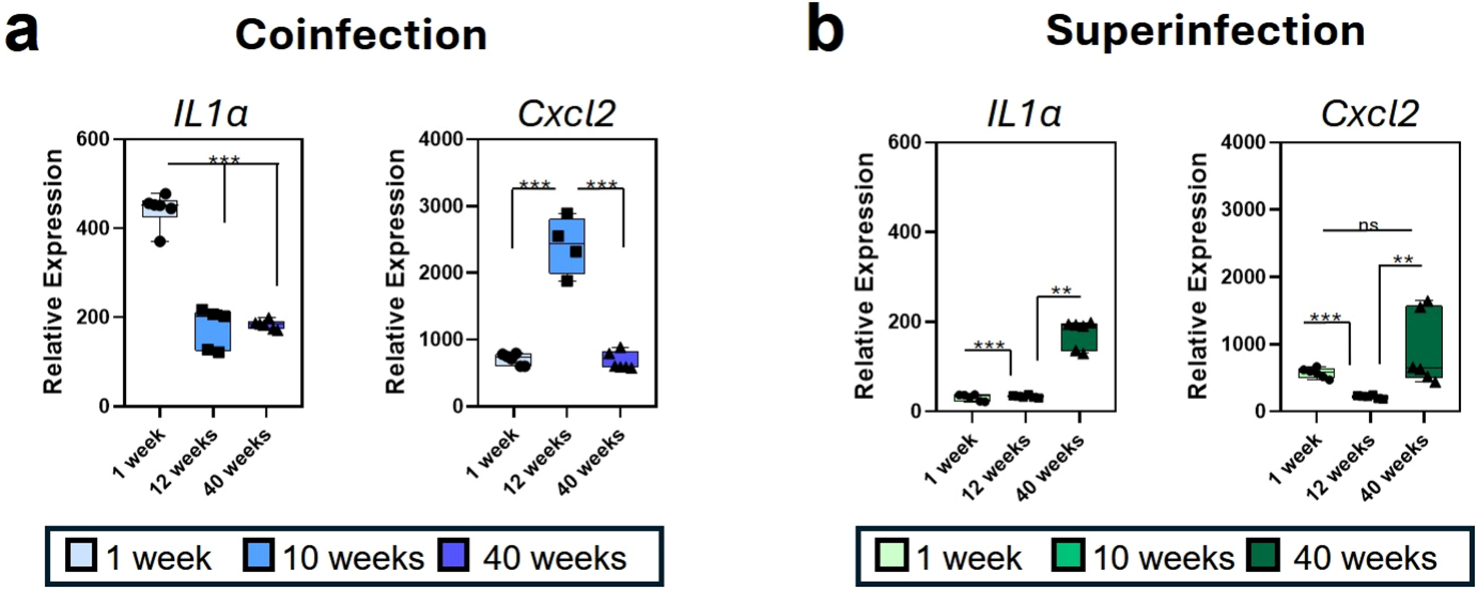
BMDM differentiated from mice of different ages exhibit different responses to coinfection and superinfection. Relative gene expression by RT-qPCR of *Il1a* and *Cxcl2* genes in BMDMs differentiated with M-CSF after coinfection with IAV and *S. pneumoniae***(a)**, or superinfection **(b)** conditions. All comparisons were made against controls of the same age. Statistical significance assessed by one-way ANOVA. **p ≤ 0.01, ***p ≤ 0.001.

The differentiation protocol using M-CSF allows for studying different aspects of innate immunity, including sequential stimulation with different PAMPs. We have recently published a differentiation protocol with M-CSF to study trained immunity *in vitro* (34). We explored this protocol to investigate trained immunity during IAV-Spn sequential infection; however, the conditions required to assess primed macrophages were not fully met, and Influenza−infected macrophages did not tolerate the prolonged incubation and resting periods demanded by the protocol. In this context and based on the similar gene expression responses analyzed in GM-CSF- and M-CSF-differentiated BMDM in coinfection and superinfection, we next differentiated BMDMs with M-CSF for 7 days and infected them with Influenza virus and *S. pneumoniae* to evaluate how aging affects the response (**Figure 4**). We measured the relative expression of *Il1a* and *Cxcl2* across BMDM extracted from three age groups of mice (1 week, 12 weeks, 40 weeks) in the coinfection and superinfection conditions using PR8 and Spn.

During coinfection, *Il1a* expression was significantly upregulated in newborns (1-week mice) as compared to adult (12 weeks) and aged (40 weeks) mice. In contrast, the expression of the chemokine *Cxcl2* was significantly higher in adult mice (12 weeks) as compared to 1-week-old and 40-week-old mice. Under superinfection conditions, *Il1a* gene expression was significantly upregulated in 40-week-old mice compared to both 1-week-old and 12-week-old mice, while *Cxcl2* was upregulated in both newborn and aged mice relative to adults, as compared to 12-week-old mice.

## Discussion

The severe morbidity and mortality associated with Influenza A virus infection are frequently driven by secondary bacterial pneumonia, where an excessive inflammatory response leads to significant tissue damage (35, 36). Macrophages are central orchestrators of this process, mediating both pathogen clearance and immunopathology (37). While tissue-resident alveolar macrophages (TR-AMs), which arise from fetal monocytes and are maintained through self-renewal in homeostasis, form the first line of defense, they are often depleted during severe influenza and replaced by recruited monocyte-derived macrophages that shape the inflammatory milieu (38–41). Our *in vitro* model, which utilizes bone-marrow-derived macrophages (BMDMs) differentiated with GM-CSF, was designed to simulate the functional response of the “second wave” of circulating Ly6C+ monocytes from the bone marrow that infiltrate the lung following influenza-induced depletion of resident alveolar macrophages (5, 6). These cells differentiate *in situ* into monocyte-derived alveolar macrophages, a population with distinct functional and transcriptional profiles that transiently dominate the alveolar space and orchestrate both the inflammatory and reparative phases of recovery (6, 42). Although the cellular crosstalk and anatomical complexity of the *in vivo* lung environment cannot be fully recapitulated by this reductionist system, it nonetheless offers a controlled platform to dissect how macrophage transcriptional programming is shaped by the timing of pathogenic encounters. Future studies employing primary human alveolar macrophages will be essential to strengthen our understanding of the potential and limitations of *in vitro* macrophage models for investigating the inflammatory properties of these key lung-resident cells. Together with the porcine alveolar macrophage data, these findings support the generalizability of our mechanistic model across host species and reinforce that infection timing and the idea of the relevance of simultaneous versus sequential, are the primary determinants of macrophage programming in shaping their response against pathogens, in this case, IAV and *Streptococcus* sp.

Our study dissects this complex interaction by demonstrating that the macrophage response is fundamentally determined by the timing of bacterial exposure relative to viral infection. This creates two immunologically distinct scenarios, each driven by unique molecular programs: simultaneous coinfection and sequential superinfection. Understanding these core responses is essential, since no studies to date have systematically analyzed the different macrophage activation states in coinfection versus superinfection scenarios.

The defining variable of coinfection (**Figure 1**) and superinfection (**Figure 2**) *in vitro* models is the timing of bacterial exposure relative to viral infection, which critically shapes macrophage responses. Simultaneous exposure (coinfection) or delayed bacterial challenge after viral priming (superinfection) leads to markedly distinct transcriptional profiles. The selection of the delayed time (48h) was based on maximizing the time between infections while still maintaining cellular viability (data not shown).

GM-CSF–differentiated BMDMs can mount an antiviral transcriptional program 8h after Influenza virus infection alone (**Figure 1**), including the upregulation of interferon-stimulated genes (ISGs). In contrast, bacterial infection triggers a broader and stronger proinflammatory transcriptional profile. When both pathogens infect simultaneously, BMDMs activate a synergistic transcriptional program, more similar to the program against bacterial infection, but that results in an amplified immune response that exceeds the effect of either pathogen alone for several genes. Regulon activity analysis (*DoRothEA*) and protein–protein interaction networks (STRING) indicate that NF-κB–dependent genes were strongly associated with this synergy. This finding suggests that in a coinfection scenario, the host response is geared towards an immediate, bacterially driven response, which may explain the rapid clinical deterioration observed in patients with concurrent infections (35).

However, when the bacterial infection occurs 48h after the initial Influenza virus infection (**Figure 2**), the transcriptional response is highly determined by the preceding viral infection. Macrophage response is apparently in a more advanced state, exhibiting higher regulation of genes involved in chemotaxis and cytokine response. This is reflected by an increase in the expression of genes such as Fibroblast Growth Factor Receptor 1 (*FGFR1*) related to positive regulation of cell population proliferation and tissue repair, often necessary after severe viral injury (43, 44) in the Influenza mock condition (48h + 8h of only Influenza infection), compared to the PR8 condition at 48h (**Figure 2c**).

Additionally, elevated expression of genes related to sulfur metabolism (such as *Ethe1*), which functionally reflects an essential compensatory mechanism for the macrophages to manage the combined oxidative stress caused by potential residual viral debris and the newly introduced bacteria, while simultaneously preserving cellular viability necessary for its pro-reparative (M2-like) functions (45). Also, at this time point (48h + 8h), there is a decrease in cytokine response (*e.g*. *Ccl22*, *Cxcl10* or *Ccl7*) and genes that have been associated with the fibrin clot formation process (*e.g*. *F13a1*). Nonetheless, when Spn is introduced after 48 hours of Influenza virus infection, all these genes are not only upregulated but synergistically elevated compared to Influenza virus infection alone (and also Spn alone) at both 48h and 48 + 8h.

The fibrin clot formation process provides a direct cellular and molecular mechanism underpinning *in vivo* observations where post-influenza, bacterial superinfection is linked to extensive thrombosis and activation of the coagulation pathway, a key contributor to severe lung pathology (46). Our data provides a potential mechanistic basis for these clinical phenomena, suggesting that viral priming renders macrophages hyper-responsive to subsequent bacterial stimuli in a manner that could promote fibrin deposition. These results are consistent with *in vivo* findings from Kathie-Anne Walters et al. (46). Their study on 1918 H1N1 and *Streptococcus pneumoniae* superinfection (72h after Influenza infection) in mice showed extensive activation of the coagulation pathway, leading to widespread thrombosis.

One limitation of this study might be the differing kinetics of protein synthesis, as each protein is produced at varying rates. Protein expression often lags behind RNA changes due to synthesis and secretion kinetics, so our collection times (8h for coinfection; 48h or 48h+8h for superinfection) may miss slower responses. While RNAseq revealed significant transcriptional shifts, protein-level differences were less pronounced. Olink proteomics aligned with RNA trends and added functional insight, but confirming synergistic protein overexpression patterns requires finer kinetic resolution.

The nature of the host response is further modulated by the specific pathogens involved. By comparing the influence of different bacterial species in secondary infection after Influenza, variations were observed across *Streptococcus* sp., but also among different *S. suis* serotypes, a main pathogen in pigs or swine PAMs (**Figure 3**). Comparable findings have been reported in previous studies examining the heterogeneity of *S. suis*–macrophage interactions (47). Zhu et al. (2024) showed that even strains of the same *S. suis* serotype, but different clonal complexes, significantly differ in their association with monocytes, complement activation, and survival in porcine blood, indicating that virulence is determined beyond the capsule type itself. Other analyses also suggest that macrophage responses vary not only across species but also among *S. suis* serotypes (48). Our findings suggest that BMDMs adaptively modulate their responses to different bacterial pathogens that are not just determined at the species level and may differ by serotype of the secondary streptococcal infection, and are less dependent on the Influenza subtype.

However, the emergence of highly pathogenic avian Influenza subtypes such as H5N1 may generate alternative gene response scenarios. Highly pathogenic avian Influenza H5N1 clade 2.3.4.4b presents high mortality in poultry and has also produced sporadic fatal human cases. In this context, viral adaptation and the specific innate−sensing machinery of each host species (avian, swine, or human) can markedly influence how the virus is detected and can shape and determine the relevance of the Influenza virus infection (49).

The relative expression of synergistic genes identified for Spn varied significantly across infections by different *Streptococcus* species (**Figure 3a**). This highlights that host-pathogen interactions are not generic and macrophage-mediated inflammatory responses depend on both pathogen and host species. Although the *S. suis* serotypes used differ in their tissue tropism (one typically associated with lung infections and the other with neuroinvasive disease), no distinct response pattern consistent with these phenotypes was observed in our model.

Monocyte differentiation shapes macrophage responses; however, in the coinfection and superinfection models presented here, the expression of the analyzed marker genes remained unchanged despite clear differentiation patterns confirmed by cytometry (**Supplementary Figure 5**). Future studies on both *in vitro* GM-CSF and M-CSF-stimulated macrophages could further elucidate superinfection-specific differences.

The characterization of M-CSF-driven macrophage differentiation, typically associated with a homeostatic context (50), leads to further exploration into how age-related factors might influence this profile during coinfection and superinfection contexts. Age is a critical determinant of immune responsiveness to influenza and secondary bacterial infections, shaping macrophage behavior and increasing susceptibility and altered inflammatory profiles in young infants (0–4 years) and elderly individuals present the groups with increased risk of severe acute respiratory infections. Analyzing the specific coinfection and superinfection responses in these groups can help to understand and prevent the disease burden.

Analysis of M-CSF differentiated macrophages from 1-, 12-, or 40-week-old mice enabled us to explore age-specific variations in macrophage responses to different types of Influenza infection. Distinct patterns of inflammatory gene expression in response to both coinfection and superinfection models revealed age-dependent gene expression differences, suggesting that macrophage responsiveness to infection can be shaped by the host age.

The *in vitro* models presented here provide simple frameworks to simulate potential aspects of *in vivo* responses to diverse Influenza infection scenarios of influenza-bacterial co-pathogenesis and underscore how pathogen identity, host age, and the timing of infection intersect to determine disease outcome. They offer controlled conditions to dissect host-pathogen interactions and immune dynamics, helping to interpret the potential severity of influenza complications and deepen our understanding of host responses across infection contexts.

## Data Availability

The datasets presented in this study can be found in online repositories. The names of the repository/repositories and accession number(s) can be found below: https://www.ncbi.nlm.nih.gov/, PRJNA1346689.

## Supplementary material

Supplementary Figure 1 Synergistic gene analysis in coinfection. **(a)** Heatmaps of the top 50 genes of clusters 2,3 and 4 from the upregulated synergistic genes in **Figure 1e**, where gene IDs are shown on the right. **(b)** KEGG pathways and GO of a list of genes with the sum of all the genes in clusters 2,3 and 4 from **Figure 1e**. **(c)** Volcano Plots displaying differentially expressed genes stimulated by PR8, Spn or coinfection. Upregulated genes are shown in red and downregulated genes in blue. Non-significant genes are displayed in grey. **(d)** GSEA analysis plot displaying top 10 significantly upregulated pathways in coinfected samples compared to Spn infected samples **(e)** Correlation plot of coinfection Log_2_FC vs *S. pneumoniae* Log_2_FC. Colored dots represent synergistically upregulated (red) or downregulated (green) genes. **(f)** PCA analysis of samples in the proteomic analysis. Individual virus and bacteria inoculations are used as comparison for the coinfection. Mock: pathogen−free medium control; single−infection controls: IAV−only or Spn−only.

Supplementary Figure 2 Synergistic gene analysis in superinfection. **(a)** PCA analysis of samples in the RNAseq analysis, and a correlation plot showing the distance between samples. **(b)** GO clustergram of synergistic genes in superinfection. **(c)** Netplot graph of the top GO functions in superinfection vs control condition. **(d)** Correlation plot of superinfection Log_2_FC vs PR8 Log_2_FC. Colored dots represent synergistically upregulated (red) or downregulated (green) genes. **(e)** PCA analysis of samples in the proteomic analysis. Individual virus and bacteria inoculations are used as a comparison. Mock: pathogen−free medium control; single−infection controls: IAV−only or Spn−only.

Supplementary Figure 3 Effects of *Streptococcus* sp. and swine Influenza subtypes on inflammatory gene expression in BMDMs. Relative *Ifnb1*, *Il6*, *Il1b* expression in BMDMs coinfected with **(a)** different *S. suis* serotypes or **(b)** four swine Influenza subtypes (H1N1, H1N2, H3N1, H3N2). No subtype pooling occurred.

Supplementary Figure 4 M-CSF differentiated BMDMs also exhibit higher inflammatory cytokine and chemokine production in coinfection and superinfection compared to individual infections. Relative gene expression by RT-qPCR of *Il6, Il1a, Il1b* genes and *Cxcl2, Ccl3*, and *Cxcl10* chemokines in BMDMs differentiated with M-CSF after coinfection **(a)** or superinfection **(b)** protocols. Statistical significance assessed by one-way ANOVA. * p ≤ 0.05, ** p ≤ 0.01, *** p ≤ 0.001.

Supplementary Figure 5 Representative flow cytometry analysis of cytokine-induced myeloid differentiation from bone marrow cells. Bone marrow (BM) cells were analyzed by flow cytometry at day 0 (top row) or after 6 days of *in vitro* differentiation with GM-CSF (middle row) or M-CSF (bottom row). The gating strategy shown involved sequential selection of viable (L/D^−^) CD45^+^ leukocytes, followed by CD11b^+^ myeloid cells. Final plots (Ly6C vs. Ly6G) identify Monocytes/Macrophages (MO) and Neutrophils (Neu). Percentages indicate the frequency of cells within the displayed gates, showing the distinct differentiation patterns induced by (favoring Neutrophils) and (favoring Monocytes/Macrophages).

Supplementary Table 1 Streptococcus suis serotypes summary. Data includes ID, origin of extraction, serotype and presence/absence of common virulence factors.

Supplementary Table 2 List of primers used for qPCR.
