# Supplementary figures and images for: Divergent macrophage responses to Influenza A virus and Streptococcus pneumoniae: co-infection drives bacterial dominance whereas superinfection favors viral priming
Source: Front Immunol. 2026 Mar 4;17:1729086. doi: 10.3389/fimmu.2026.1729086 (PMC12996220; doi:10.3389/fimmu.2026.1729086)

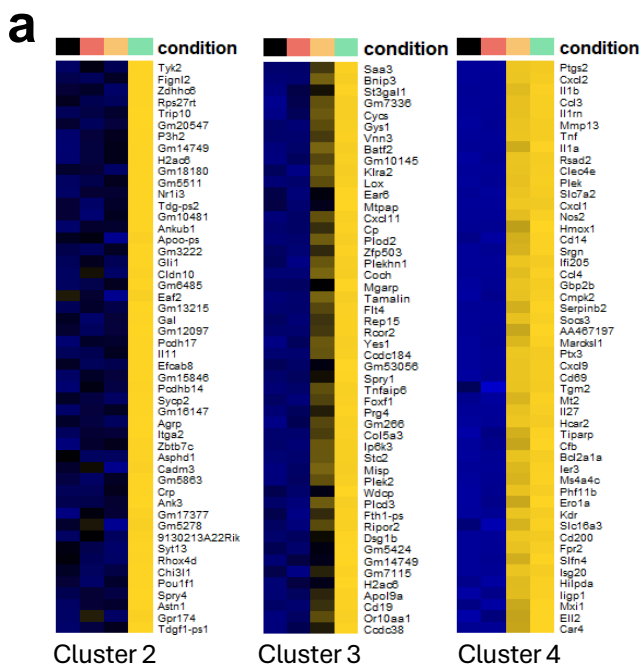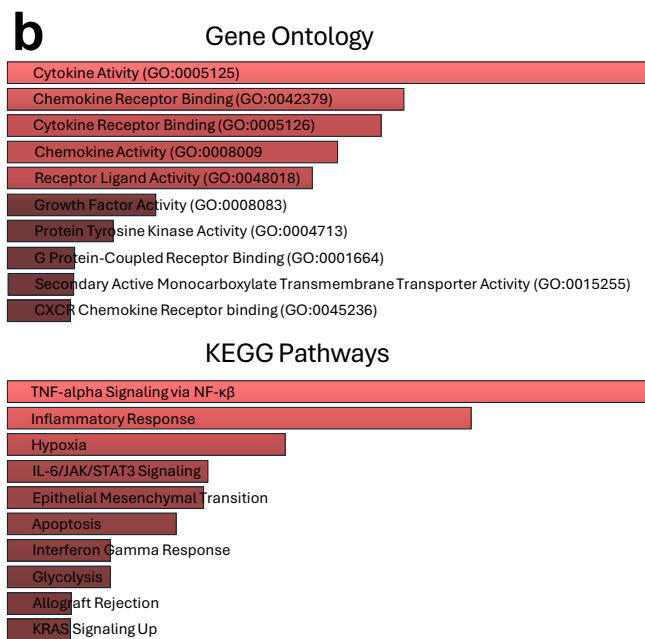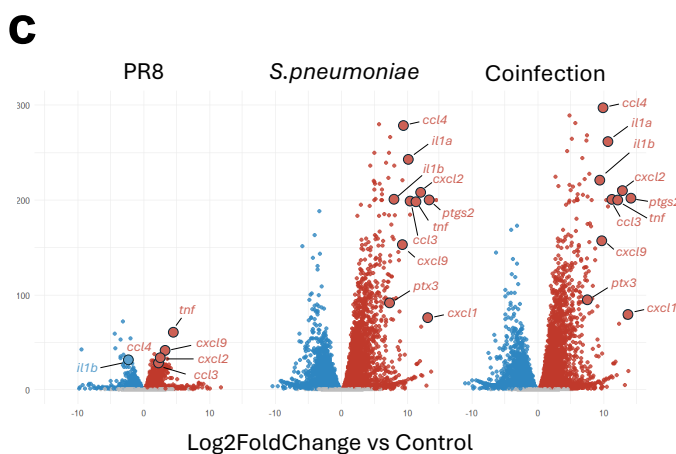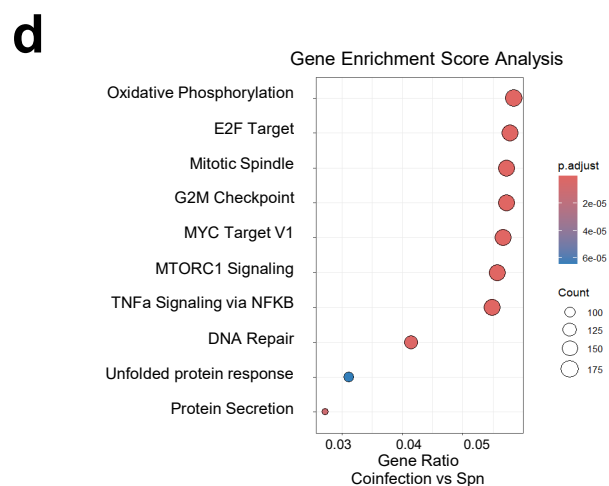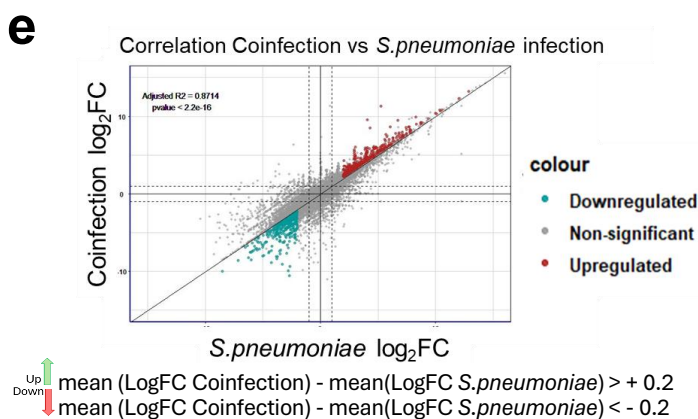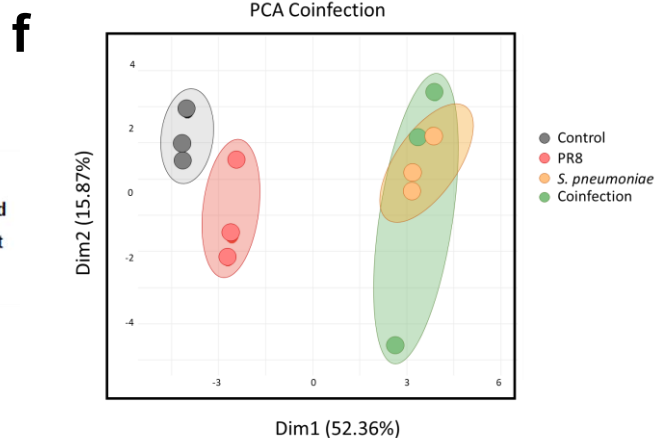

Supplement: Supplementary Figure 1 — Synergistic gene analysis in coinfection. (a) Heatmaps of the top 50 genes of clusters 2,3 and 4 from the upregulated synergistic genes in Figure 1e, where gene IDs are shown on the right. (b) KEGG pathways and GO of a list of genes with the sum of all the genes in clusters 2,3 and 4 from Figure 1e. (c) Volcano Plots displaying differentially expressed genes stimulated by PR8, Spn or coinfection. Upregulated genes are shown in red and downregulated genes in blue. Non-significant genes are displayed in grey. (d) GSEA analysis plot displaying top 10 significantly upregulated pathways in coinfected samples compared to Spn infected samples (e) Correlation plot of coinfection Log2FC vs S. pneumoniae Log2FC. Colored dots represent synergistically upregulated (red) or downregulated (green) genes. (f) PCA analysis of samples in the proteomic analysis. Individual virus and bacteria inoculations are used as comparison for the coinfection. Mock: pathogen−free medium control; single−infection controls: IAV−only or Spn−only. [file DataSheet1.pdf]

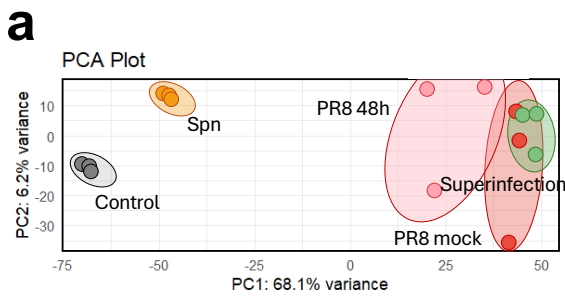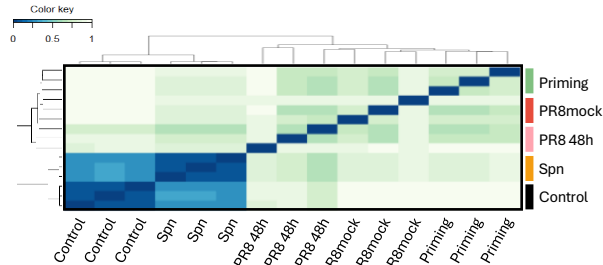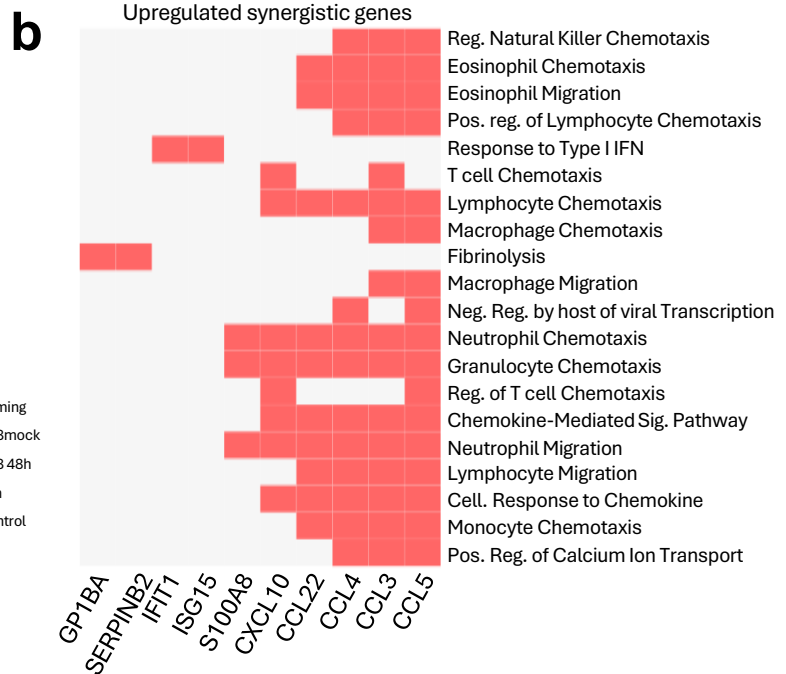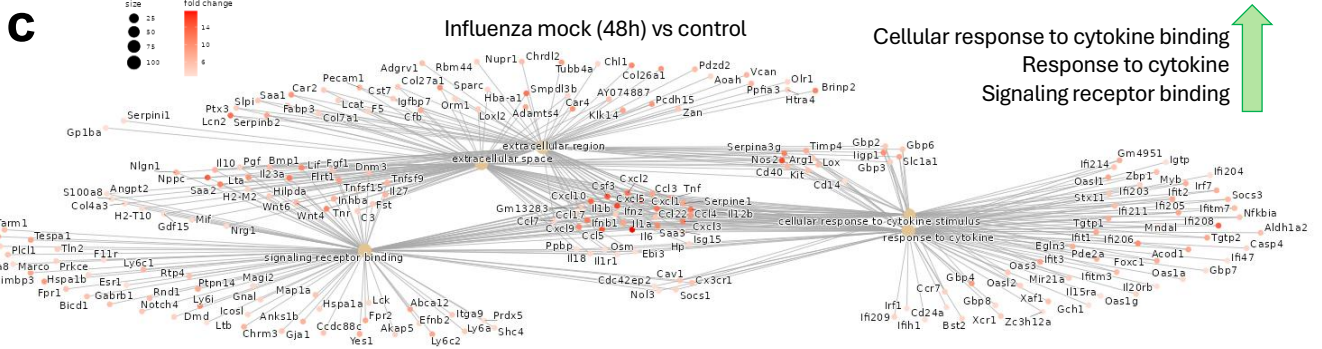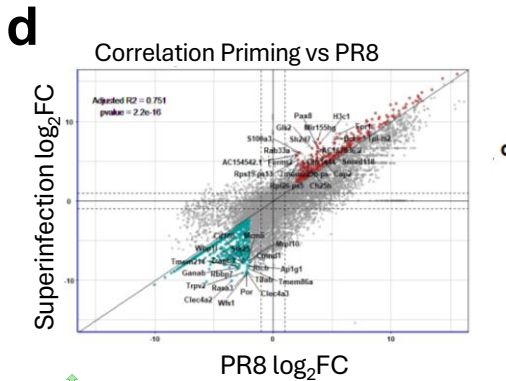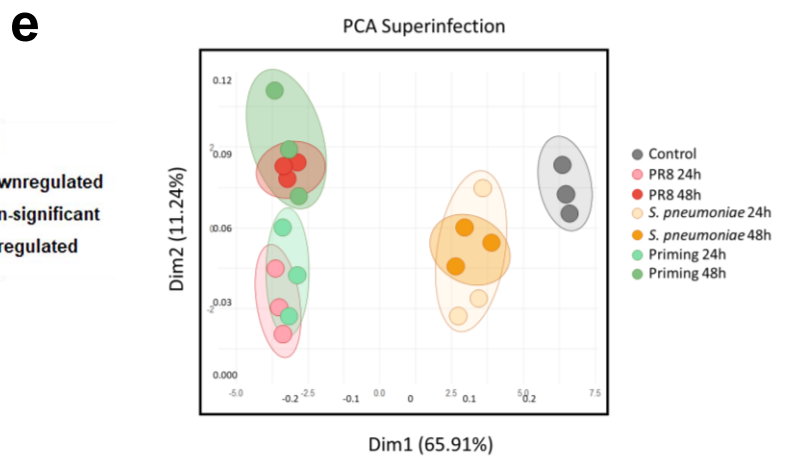

Supplement: Supplementary Figure 2 — Synergistic gene analysis in superinfection. (a) PCA analysis of samples in the RNAseq analysis, and a correlation plot showing the distance between samples. (b) GO clustergram of synergistic genes in superinfection. (c) Netplot graph of the top GO functions in superinfection vs control condition. (d) Correlation plot of superinfection Log2FC vs PR8 Log2FC. Colored dots represent synergistically upregulated (red) or downregulated (green) genes. (e) PCA analysis of samples in the proteomic analysis. Individual virus and bacteria inoculations are used as a comparison. Mock: pathogen−free medium control; single−infection controls: IAV−only or Spn−only. [file DataSheet2.pdf]

**a**

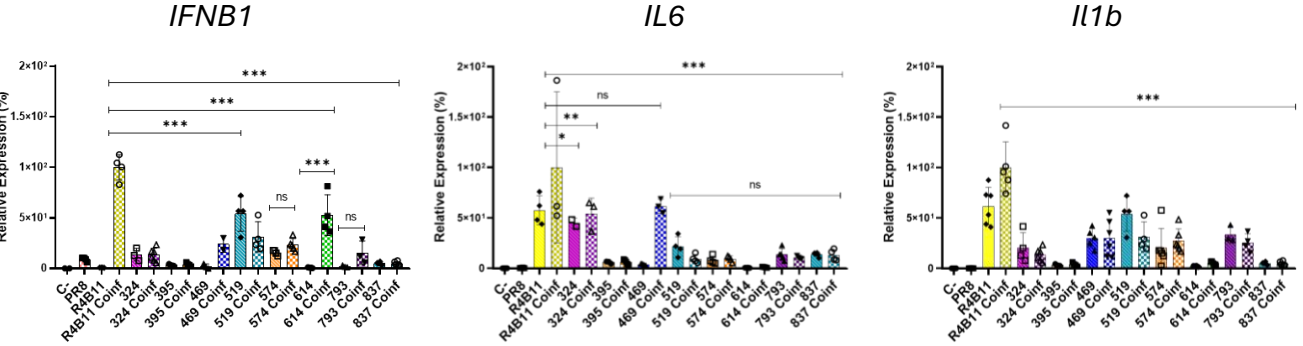

**b**

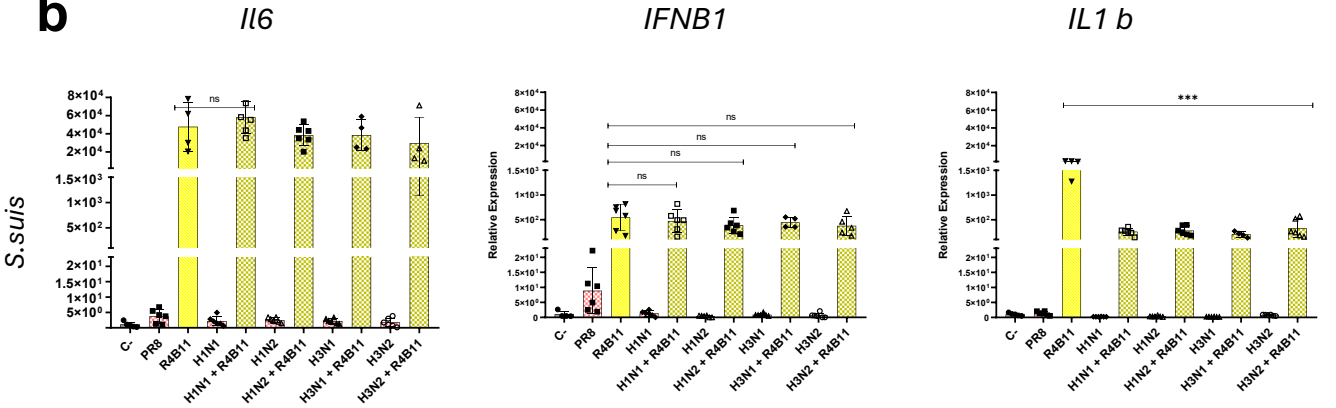

Supplement: Supplementary Figure 3 — Effects of Streptococcus sp. and swine Influenza subtypes on inflammatory gene expression in BMDMs. Relative Ifnb1, Il6, Il1b expression in BMDMs coinfected with (a) different S. suis serotypes or (b) four swine Influenza subtypes (H1N1, H1N2, H3N1, H3N2). No subtype pooling occurred. [file DataSheet3.pdf]

**a** Coinfection

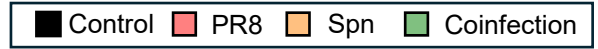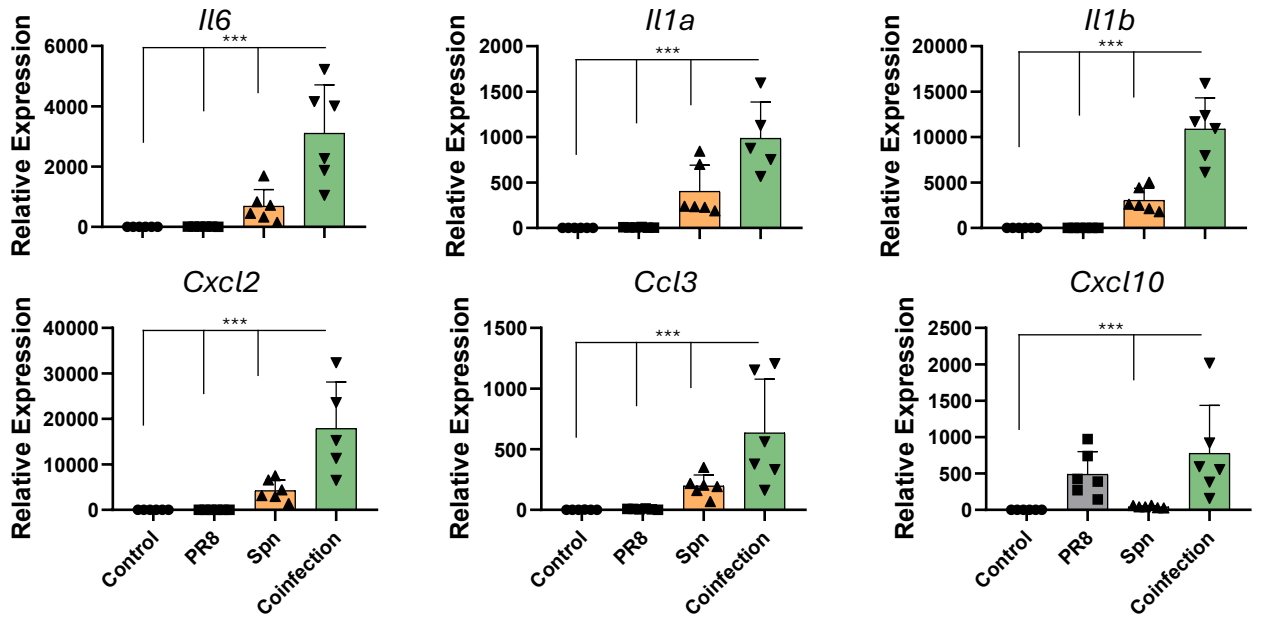

**b** Superinfection

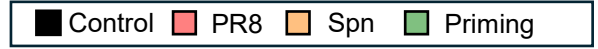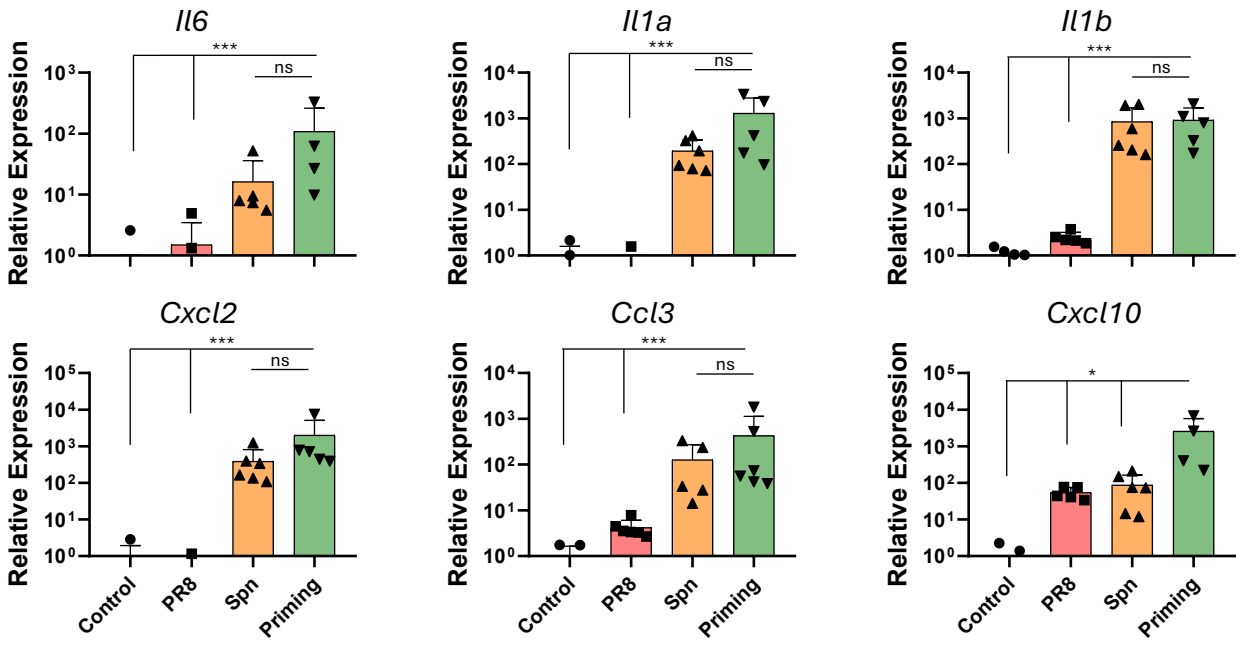

Supplement: Supplementary Figure 4 — M-CSF differentiated BMDMs also exhibit higher inflammatory cytokine and chemokine production in coinfection and superinfection compared to individual infections. Relative gene expression by RT-qPCR of Il6, Il1a, Il1b genes and Cxcl2, Ccl3, and Cxcl10 chemokines in BMDMs differentiated with M-CSF after coinfection (a) or superinfection (b) protocols. Statistical significance assessed by one-way ANOVA. * p ≤ 0.05, ** p ≤ 0.01, *** p ≤ 0.001. [file DataSheet4.pdf]

a

Bm total  
(do)

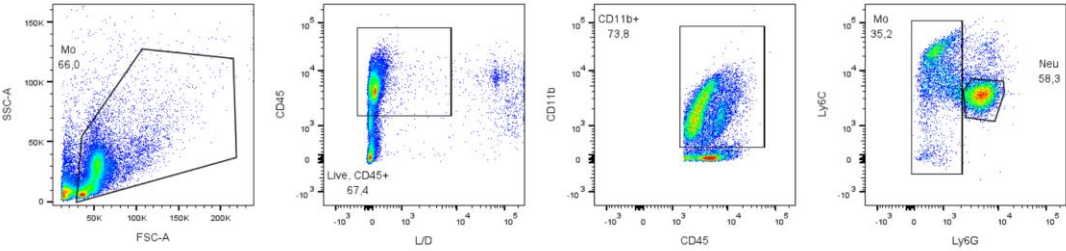

GM-CSF  
(d6)

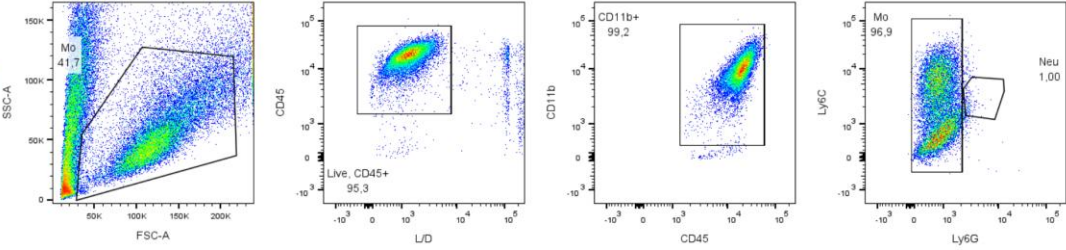

M-CSF  
(d6)

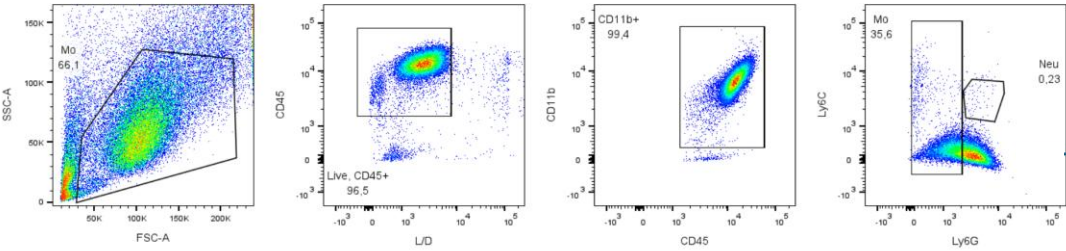

Supplement: Supplementary Figure 5 — Representative flow cytometry analysis of cytokine-induced myeloid differentiation from bone marrow cells. Bone marrow (BM) cells were analyzed by flow cytometry at day 0 (top row) or after 6 days of in vitro differentiation with GM-CSF (middle row) or M-CSF (bottom row). The gating strategy shown involved sequential selection of viable (L/D−) CD45+ leukocytes, followed by CD11b+ myeloid cells. Final plots (Ly6C vs. Ly6G) identify Monocytes/Macrophages (MO) and Neutrophils (Neu). Percentages indicate the frequency of cells within the displayed gates, showing the distinct differentiation patterns induced by (favoring Neutrophils) and (favoring Monocytes/Macrophages). [file DataSheet5.pdf]
